# Supplementary material for: Epithelial-Myeloid cell crosstalk regulates acinar cell plasticity and pancreatic remodeling in mice
Source: eLife. 2017 Oct 5;6:e27388. doi: 10.7554/eLife.27388 (PMC5690281; doi:10.7554/eLife.27388)
Supplement: Supplementary file 1. [file elife-27388-supp1.docx]

**Supplementary file 1: Primary antibodies used in this study**

| **Immunostaining Antibody** | **Supplier** | **Catalog**  **Number** | **IHC dilution** | **IF Dilution** |
| --- | --- | --- | --- | --- |
| Alpha-smooth muscle actin | Sigma-Aldrich | A2547 | - | 1:1000 |
| Amylase | Sigma-Aldrich | A8273 | - | 1:100 |
| CD8 | Cell Signaling | 98941S | - | 1:300 |
| CK19 (TromaⅢ) | Iowa Developmental Hybridoma Bank | - | 1:100 | 1:100 |
| Cleaved Caspase-3 | Cell Signaling | 9661 | 1:400 | - |
| E-Cadherin | BD Biosciences | 610181 | - | 1:100 |
| F4/80 | BMA Biomedicals | T-2006 | 1:100 | - |
| GFP | Abcam | Ab6673 | 1:200 | - |
| Ki67 | Vector Laboratories | VP-RM04 | 1:100 | - |
| Mist1 | - | - | 1:400 | 1:400 |
| PDGF Receptor beta | Abcam | Ab32570 | - | 1:200 |
| p-EGFR(Tyr1068) | Cell Signaling | 3777 | 1:100 | - |
| p-ERK1/2 (phospho-p44/42) | Cell Signaling | 4370 | 1:100 | 1:100 |
| p-ERK1/2 (phospho-p44/42) | ThermoFisher Scientific | 14-9109 | - | 1:100 |
| Vimentin | Cell Signaling | 5741 | - | 1:200 |
| **Western-blot Antibody** | **Supplier** | **Catalog Number** | **Dilution** |  |
| Collagen I | Abcam | ab34710 | 1:1000 |  |
| β-Actin | Santa Cruz | sc-69879 | 1:1000 |  |
| MMP2 | ThermoFisher Scientific | PA1-16667 | 1:2000 |  |
| **Flow Cytometry Antibody** | **Supplier** | **Clone** | **Dilution** |  |
| CD3 | BD Pharmingen | 17A2 | 1:50 |  |
| CD11b | BD Pharmingen | M1/70 | 1:50 |  |
| CD11c | BD Pharmingen | HL3 | 1:50 |  |
| CD31 | BD Pharmingen | MEC 13.3 | 1:50 |  |
| CD45 | Invitrogen | 30-F11 | 1:50 |  |
| CD64 | BD Pharmingen | X54-5/7.1.1 | 1:50 |  |
| CD206 | AbD Serotec | MR5D3 | 1:50 |  |
| F4/80 | BD Pharmingen | BM8 | 1:50 |  |
| Gr-1 | BD Pharmingen | RB6-8C5 | 1:50 |  |
