# Supplementary material for: Epithelial-Myeloid cell crosstalk regulates acinar cell plasticity and pancreatic remodeling in mice
Source: eLife. 2017 Oct 5;6:e27388. doi: 10.7554/eLife.27388 (PMC5690281; doi:10.7554/eLife.27388)
Supplement: Supplementary file 2. [file elife-27388-supp2.docx]

**Supplementary file 2: Primer sequences for quantitative RT-PCR**

| **Gene** | **Forward Primer** | **Reverse Primer** |
| --- | --- | --- |
| *Areg* | GGTCTTAGGCTCAGGCCATTA | CGCTTATGGTGGAAACCTCTC |
| *Arg1* | CTCCAAGCCAAAGTCCTTAGAG | AGGAGCTGTCATTAGGGACATC |
| *Col1a1* | GCTCCTCTTAGGGGCCACT | CCACGTCTCACCATTGGGG |
| *Col3a1* | CTGTAACATGGAAACTGGGGAAA | CCATAGCTGAACTGAAAACCACC |
| *Cyclophilin A* | TCACAGAATTATTCCAGGATTCATG | TGCCGCCAGTGCCATT |
| *Egf* | AGCATCTCTCGGATTGACCCA | CCTGTCCCGTTAAGGAAAACTCT |
| *Egfr* | GCCATCTGGGCCAAAGATACC | GTCTTCGCATGAATAGGCCAAT |
| *Ereg* | CTGCCTCTTGGGTCTTGACG | GCGGTACAGTTATCCTCGGATTC |
| *Fn1* | ATGTGGACCCCTCCTGATAGT | GCCCAGTGATTTCAGCAAAGG |
| *Chil3* | CAGGTCTGGCAATTCTTCTGAA | GTCTTGCTCATGTGTGTAAGTGA |
| *Csf2* | ATGCCTGTCACGTTGAATGAAG | GCGGGTCTGCACACATGTTA |
| *Hbegf* | CGGGGAGTGCAGATACCTG | TTCTCCACTGGTAGAGTCAGC |
| *Il1β* | GTGGCTGTGGAGAAGCTGTG | GAAGGTCCACGGGAAAGACAC |
| *Il6* | TTCCATCCAGTTGCCTTCTTGG | TTCTCATTTCCACGATTTCCCAG |
| *Mmp2* | CAAGTTCCCCGGCGATGTC | TTCTGGTCAAGGTCACCTGTC |
| *Mmp7* | GGAGATGCTCACTTTGACAAGGA | ATTCATGGGTGGCAGCAAAC |
| *Mmp9* | CTGGACAGCCAGACACTAAAG | CTCGCGGCAAGTCTTCAGAG |
| *Mmp12* | GAGTCCAGCCACCAACATTAC | GCGAAGTGGGTCAAAGACAG |
| *Mmp14* | CAGTATGGCTACCTACCTCCAG | GCCTTGCCTGTCACTTGTAAA |
| *Mrc1* | CTCTGTTCAGCTATTGGACGC | CGGAATTTCTGGGATTCAGCTTC |
| *Msr1* | GCACAATCTGTGATGATCGCT | CCCAGCATCTTCTGAATGTGAA |
| *Shh* | CAAAGCTCACATCCACTGTTCTG | GAAACAGCCGCCGGATTT |
| *Tgfα* | CACTCTGGGTACGTGGGTG | CACAGGTGATAATGAGGACAGC |
| *Tgfβ1* | TGACGTCACTGGAGTTGTACGG | GGTTCATGTCATGGATGGTGC |
| *Tnfα* | CATCTTCTCAAAATTCGAGTGACAA | TGGGAGTAGACAAGGTACAACCC |
| *Transgenic Kras* | CAAGGACAAGGTGTACAGTTATGTGACT | GCCTGCGACGGCGGCATCTGC |
