# Supplementary material for: Epithelial-Myeloid cell crosstalk regulates acinar cell plasticity and pancreatic remodeling in mice
Source: eLife. 2017 Oct 5;6:e27388. doi: 10.7554/eLife.27388 (PMC5690281; doi:10.7554/eLife.27388)
Supplement: Transparent reporting form [file elife-27388-transrepform.pdf]

eLIFE
1st Floor
24 Hills Road
Cambridge CB2 1JP, UK
P 01223 855340
W elifesciences.org
T @elife

eLife's transparent reporting form

We encourage authors to provide detailed information *within their submission* to facilitate the interpretation and replication of experiments. If you have any questions, please contact us: editorial@elifesciences.org.

Sample-size estimation

- You should state whether an appropriate sample size was computed when the study was being designed
- You should state the statistical method of sample size computation and any required assumptions
- If no explicit power analysis was used, you should describe how you decided what sample (replicate) size (number) to use

Please outline where this information can be found within the submission (e.g., page numbers or figure legends), or explain why this information doesn't apply to your submission:

eLife Sciences Publications, Ltd is a limited liability non-profit non-stock corporation incorporated in the State of Delaware, USA, with company number 5030732, and is registered in the UK with company number FC030576 and branch number BR015634 at the address 1st Floor, 24 Hills Road, Cambridge CB2 1JP | August 2014

At the planning stage, we based our expectations on similar experiments previously performed in our laboratory, as well as in the literature. For the experimental cohorts, we used 4 to 8 mice per genotype per time point. The number of animals used for each experiment is specified in the figure legends. For quantification studies, we reviewed the histology from all the animals and 3 samples were used for in depth analysis.

Biological replicates of the experiments are a necessity of this kind of study, as typically only a small number of mice with the appropriate genotype reach the correct age at any given point. Control animals were included in each of the biological replicates. Thus, for example, the iKras* control mice in figure 4B are littermates of the iKras*;CD11b-DTR mice in the same figure, even though the iKras* phenotype upon Kras inactivation was previously published by our group. All the images and data in the manuscript only refer to the mice analyzed for this study and do not include historic samples.

Each experiment typically included multiple analysis time points, and a similar number of experimental and control animals were included in each of those.

Histopathological quantification was performed on masked samples by a pathologist (Dr Wei Yan); raw data were returned to our laboratory and unmasked to generate the final graphs.

A key aspect of planning experiments such as those in the current manuscript is the inclusion of appropriate controls. To control for the presence of Kras, mice lacking the transgene were used. Whenever possible, Ptf1a-Cre mice were used as control as these animals are hemizygous for Ptf1a, a key acinar transcription factor; loss of one copy of Ptf1a predisposes the mice to Kras-induced transformation.

Doxycycline was administered to these control animals in parallel as to the experimental animals to control for un-specific effects of this antibiotic.

For the experiments involving CD11b-DTR mice, iKras* mice lacking this allele were treated with Diphtheria Toxin to control for any non-specific effect of the toxin itself. Additionally, iKras*;CD11b-DTR not treated with DT were included as controls. Appropriate depletion of myeloid cells (or lack of depletion in the controls) was verified in each experimental animal.

Replicates

- You should report how often each experiment was performed
- You should include a definition of biological versus technical replication
- The data obtained should be provided and sufficient information should be provided to indicate the number of independent biological and/or technical replicates
- If you encountered any outliers, you should describe how these were handled
- Criteria for exclusion/inclusion of data should be clearly stated

- High-throughput sequence data should be uploaded before submission, with a private link for reviewers provided (these are available from both GEO and ArrayExpress)

Please outline where this information can be found within the submission (e.g., page numbers or figure legends), or explain why this information doesn't apply to your submission:

Each experiment includes between 3 and 5 mice; each experiment was performed usually 3 times, but at least 2 independent times, and up to 5 times. Each animal is considered a biological replicate. For qPCR analysis, each dot in the graphs represents an individual mouse (biological replicate) and the analysis was performed in duplicates (technical replicates).

Mice were excluded from analysis if the genotype failed to verify after sacrifice (e.g. no Kras expression in samples that had originally be expected to express Kras, lack of a transgenic Kras band upon re-genotype). Genotype errors are rare in our laboratory (less than 1 in 100) but occasionally occur, this our policy is to collect a fresh DNA sample whenever mice are sacrificed. RNA or flow samples were occasionally excluded if found to be of insufficient quality or contaminated (e.g. occasional contamination of pancreas samples by lymph nodes); this is an exceedingly rare event. Both of these events are mostly only applicable to wild type pancreas samples, where extraction of high quality RNA is challenging given the large amounts of RNAses in the tissue, and lymph nodes are small. Neoplastic tissue produces more stable RNA (due to decreased enzyme production) and the lymph nodes are large and easily identified.

Statistical reporting

- Statistical analysis methods should be described and justified
- Raw data should be presented in figures whenever informative to do so (typically when N per group is less than 10)
- For each experiment, you should identify the statistical tests used, exact values of N, definitions of center, methods of multiple test correction, and dispersion and precision measures (e.g., mean, median, SD, SEM, confidence intervals; and, for the major substantive results, a measure of effect size (e.g., Pearson's r, Cohen's d)
- Report exact p-values wherever possible alongside the summary statistics and 95% confidence intervals. These should be reported for all key questions and not only when the p-value is less than 0.05.

Please outline where this information can be found within the submission (e.g., page numbers or figure legends), or explain why this information doesn't apply to your submission:

Specific statistical methods used in each experiment are detailed in the figure legends. Raw data is presented in the figures, with each sample (mouse) represented by an individual dot.
Of note, compared with the initial submission, we now include specific p values for the graphs. Further, we have re-analyzed our data using two-tailed t tests for increased stringency.

(For large datasets, or papers with a very large number of statistical tests, you may upload a single table file with tests, Ns, etc., with reference to page numbers in the manuscript.)

Additional data files ("source data")

- We encourage you to upload relevant additional data files, such as numerical data that are represented as a graph in a figure, or as a summary table
- Where provided, these should be in the most useful format, and they can be uploaded as "Source data" files linked to a main figure or table
- Include model definition files including the full list of parameters used
- Include code used for data analysis (e.g., R, MatLab)
- Avoid stating that data files are "available upon request"

Please indicate the figures or tables for which source data files have been provided:

We included the raw data table for the histopathological quantification in Figure 1D. Tables for the other graphs are not included as for most of them, individual data points are provided and when we did not do so, specific p values and included in the figures.
